# Resistant Ventricular Arrhythmia and the Role of Overdrive Pacing in the Suppression of the Electrical Storm

**DOI:** 10.1155/2019/6592927

**Published:** 2019-05-22

**Authors:** Mohamed Magdi, Mahmood Mubasher, Hakam Alzaeem, Tahir Hamid

**Affiliations:** Hamad Medical Corporation, Qatar

## Abstract

Ventricular arrhythmia storm is a state of cardiac instability characterized by multiple ventricular arrhythmias or multiple ICD therapies within a 24-hour duration. Management of this life-threatening state depends on the reversal of the cause besides either electrical or medical management of the arrhythmia. We report a case of a 54-year-old male who underwent a percutaneous coronary intervention following massive acute myocardial infarction. Afterwards, he developed frequent life-threatening ventricular arrhythmias that required multiple shocks and antiarrhythmic medications. Despite all these interventions, it was very difficult to control the electrical instability, but after overdrive ventricular pacing, the storm subsided and within a few days the case was stabilized. Overdrive pacing is an easy temporary modality to control the resistant arrhythmia following myocardial infarction.

## 1. Introduction

Electrical storm is a life-threatening arrhythmia that might occur after myocardial infarction. In severe cases, arrhythmias are resistant to medications and the direct current (DC) shock. Repeated shocks are also injurious to the heart and lead to progressive myocardial inflammation and fibrosis [1–4]. The management is usually difficult and challenging with poor prognosis and high mortality. In our case, we found that temporary overdrive pacing gives a safer method to abort the arrhythmia and to avoid progressive myocardial damage with repeated electrical shocks.

## 2. Case History

A 54-year-old diabetic and hypertensive male patient presented with a central chest pain while sleeping that lasted for an hour. On the ambulance, he became drowsy and developed ventricular tachycardia that was reverted to sinus rhythm by DC shock.

**Figure 1:**
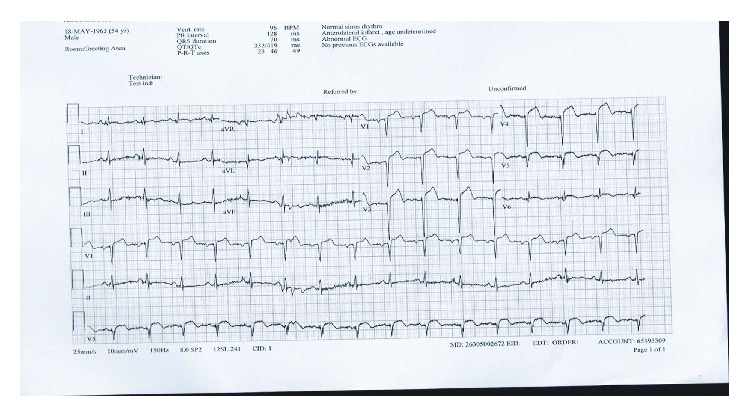
Normal sinus rhythm, ST elevation, and Q waves in leads V1, V2, V3, V4, V5, and V6.

On admission to ED (3 hours after the pain), he was in pain and in the physical exam and he was fully conscious and oriented and in distress. Vitals were within the normal limits except for a slightly elevated blood pressure of 150/90. ECG showed normal sinus rhythm and ST elevation in leads V1 to V6 with deep Q waves in the same leads (Figure 1).

In the coronary catheterization lab (30 minutes after admission to ED), coronary angiography showed total proximal left anterior descending (LAD) artery occlusion and midleft circumflex (LCX) 80% lesion. The LAD lesion was stented with a DES.

**Figure 2:**
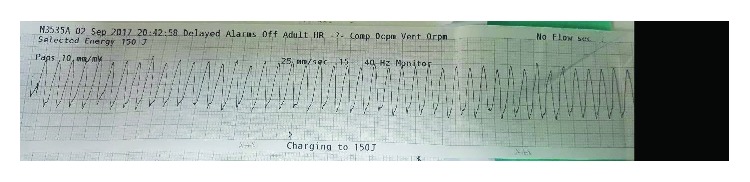
Ventricular tachycardia.

After the procedure, he was stable with residual chest pain. Then, 2 hours later, he was found unresponsive with pulseless ventricular tachycardia, and after a single DC shock, he regained his consciousness and his sinus rhythm again (Figure 2).

**Figure 3:**
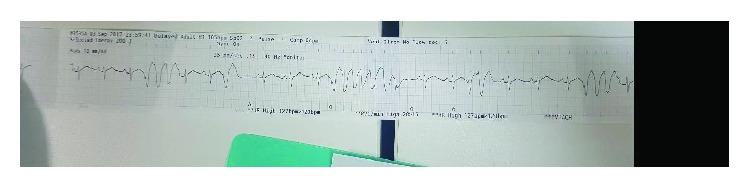
Multiple PVCs and NSVT.

The next morning, he developed asymptomatic sustained ventricular tachycardia that reverted spontaneously to sinus rhythm; then, the telemetry showed multiple premature ventricular complexes (PVCs) and nonsustained ventricular tachycardia (NSVT) (Figure 3). Amiodarone 150 mg IV bolus was given followed by 1 mg/minute IV infusion for 6 hours and then 0.5 mg/minute IV infusion for 18 hours. Serum electrolytes were within normal limits.

After two hours, he developed again asymptomatic ventricular tachycardia and IV lidocaine 70 mg bolus was given. Then, relook coronary angiography showed patent LAD stent and small ostial LCX clot that was aspirated.

**Figure 4:**
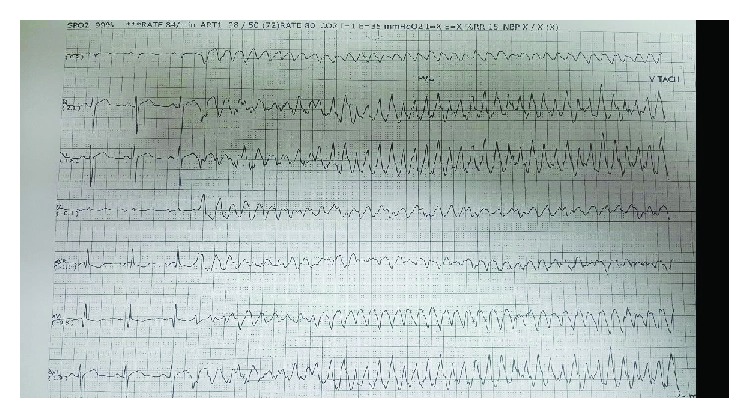
Ventricular tachycardia.

In the evening, he had ventricular tachycardia (Figure 4) with hypotension, so the situation required the suppression of the arrhythmia by a DC shock, endotracheal intubation, and sedation (midazolam 0.1 mg/kg/hour). An intra-aortic balloon pump (IABP) and norepinephrine infusion 0.1 mcg/kg/minute were initiated to support his cardiogenic shock state.

**Figure 5:**
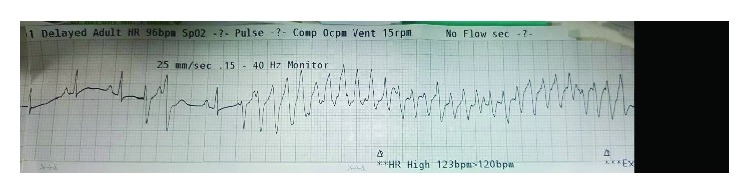
Ventricular tachycardia preceded by PVCs.

The next day, clusters of ventricular tachycardia triggered by ventricular premature contractions occurred (Figure 5) and required multiple electrical defibrillations. Consequently, the patient was transferred to the catheterization lab, and a transcutaneous pacemaker was inserted.

**Figure 6:**
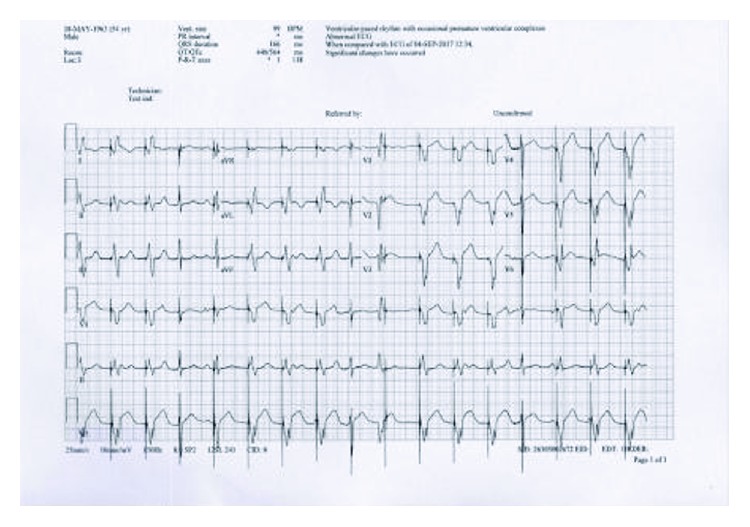
Ventricular paced rhythm at rate of 100.

After overdrive pacing with a rate of 100, his rhythm was stable with complete suppression of PVCs and no further attacks of ventricular arrhythmias (Figure 6).

In the next day, gradual weaning of inotropes and sedations started until it is completely stopped. His rhythm was stable on oral amiodarone 400 mg twice daily and metoprolol 25 mg twice daily in addition to the pacing.

**Figure 7:**
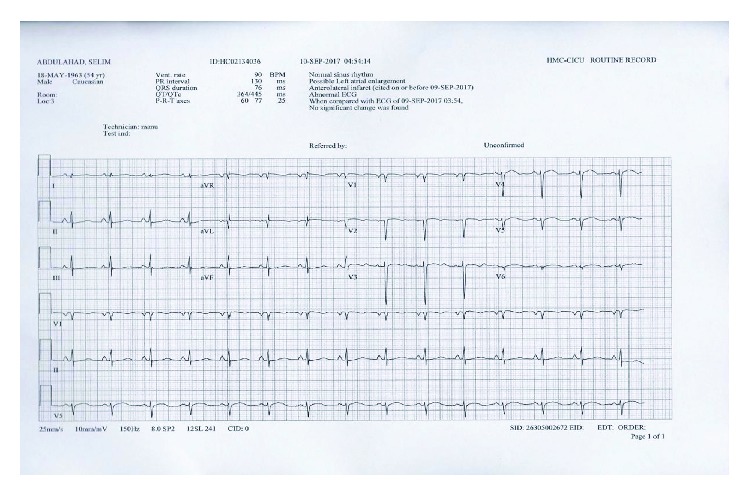
Sinus rhythm after pacemaker removal.

After another 24 hours of stable rhythm, the pacemaker was removed and close observation showed no any ventricular arrhythmias (Figure 7). The next day, the patient was weaned from the mechanical ventilator.

He was observed in the coronary care unit for 2 days showing hemodynamic and electrical stability. After 2 weeks of discharge, he was seen in the clinic in a good state of health.

## 3. Discussion

Electrical storm (ES) indicates a state of life-threatening cardiac electrical instability characterized by clusters of ventricular arrhythmias in a short amount of time. It is defined as the presence of at least 3 distinct episodes of sustained ventricular tachycardia or VF in the last 24 hours. In the patients with an ICD, the most widely accepted definition of the electrical storm is three or more appropriate therapies for ventricular tachyarrhythmias, including antitachycardia pacing or shocks, within 24 hours [5].

Studies have shown that in only 10-25% of patients with the electrical storm, clear precipitating causes were identified [6]. Some important causes are reversible, and their management can facilitate the control of the arrhythmias like acute myocardial ischemia, new or worsening heart failure, drug intoxication, or electrolyte disturbances.

ES is a clinical emergency that needs appropriate early management. Initially, antiarrhythmic drugs such as beta blocker and amiodarone are usually used [7, 8]. The drug of choice to start is amiodarone 150 mg IV over 10 minutes, followed by 1 mg/minute IV infusion for 6 hours and then by 0.5 mg/minute IV infusion for 18 additional hours.

Then, beta blockers are very important in the suppression of the sympathetic discharges after the arrhythmias and DC shocks. Propranolol (40 mg every 6 hours) has shown superior efficacy to metoprolol in a trial performed on 60 patients with electrical storm who received amiodarone, and this showed that the time to termination of the arrhythmias was earlier in the propranolol group in addition to lower rate of tachycardia recurrence or ICD discharges and shorter stay in the hospital [9]. There are other medications that can be used as an adjunct to amiodarone and propranolol like procainamide, lidocaine, sotalol, and mexiletine [10–12].

In case of failure of medical therapy, radiofrequency catheter ablation of the arrhythmogenic focus is a promising effective method for the management of the ventricular arrhythmia [13].

In the recent European Society of Cardiology's guidelines for the management of ventricular arrhythmias, transvenous catheter overdrive stimulation received a class IIa recommendation, and level of evidence C in case of recurrent ventricular arrhythmia despite the use of drug therapy and catheter ablation is not possible [14].

In our case, we report a state of severe electrical storm following a massive acute myocardial infarction. This arrhythmia persisted despite the frequent DC shocks in addition to amiodarone, lidocaine, and beta blocker therapy. In this life-threatening condition, an early intervention is needed to save life, to prevent the short- and long-term sequelae on the myocardial muscles, and to improve the patient's quality of life. Overdrive pacing is a method utilized to terminate the tachycardia by pacing the patient's heart at a rate faster than the intrinsic rhythm. Pacing the patient's heart at a rate of 100 beats per minute succeeded in controlling the arrhythmia without the need of multiple DC shocks.

Kurisu et al. [15] reported a similar case, but the electrical storm developed after 7 days of the myocardial infarction that was resistant to the conventional antiarrhythmic medications, and overdrive pacing was a feasible easy way to stabilize the case. Yoshida et al. [16] achieved an excellent suppression of the arrhythmia by overdrive pacing in a patient with multiple myeloma and coronary artery disease after coronary artery bypass grafting.

Additionally, in the management of the ES, there were other reported management plans. Mulpuru et al. [17] found that sedation with propofol was helpful in controlling the ventricular arrhythmias in a patient who presented with multiple ICD discharges and showed a poor response to oral antiarrhythmic medications. Tsagalou et al. [18] reported a case of an electrical storm that was refractory to amiodarone, metoprolol, and lignocaine, but after substitution of metoprolol with the nonselective propranolol, he achieved a good control of the electrical discharges, showing that propranolol is better than other selective beta blockers in treating ES.

ES usually reflects a poor prognosis with higher mortality, increased rate of hospitalization, and negative impact on the quality of life. Also, the electrical cardiac shocks besides their beneficial control of the arrhythmia and their frequency lead to more cardiac damage and progressive inflammation that later end up with cardiac fibrosis and worsening cardiac function [3, 4].

## 4. Conclusions

In severe cases of electrical storm that is refractory to the initial management, overdrive pacing is a helpful temporary method in the stabilization of the case and it could be a very important early tool to decrease the mortality in resistant arrhythmia after myocardial infarction.
